# The Surgical Treatment for Atrial Fibrillation: Ablation Technology and Surgical Approaches

**DOI:** 10.5041/RMMJ.10121

**Published:** 2013-07-25

**Authors:** Linda Henry, Niv Ad

**Affiliations:** Cardiac Surgery Research Department, Inova Heart and Vascular Institute, Inova Fairfax Hospital, Falls Church, VA, USA

**Keywords:** Ablation, technology, cryothermy, radiofrequency

## Abstract

The Cox maze procedure developed originally in 1987 by Dr James Cox has evolved from a “cut and sew” surgical procedure, where the maze was applied using multiple surgical cuts, to an extensive use of surgical ablation technology where ablation lesions are placed with alternative energy sources (radiofrequency, cryothermy, microwave, and high-frequency ultrasound). Furthermore, the procedure has changed from a median sternotomy approach only to one that can be performed minimally invasively and robotically. The purpose of this paper is to review the current available technology for the ablation of atrial fibrillation as well as the different procedural approaches for the surgical ablation of atrial fibrillation.

## BACKGROUND

On September 25, 1987, James L. Cox, MD at Barnes Hospital in St Louis, Missouri, performed the first maze procedure for atrial fibrillation. The original maze procedure involved cutting and sewing multiple incisions in the left and right atria to interrupt all macro re-entrant atrial fibrillation circuit options in the atria. After several modifications, this surgery is known today as the maze III procedure, and in its modern form most of the surgical incisions have been replaced by surgically placed linear lesion lines created by alternative energy sources (cryothermy, radiofrequency, and high-intensity focused ultrasound) and specially designed devices.^1^–^6^

The maze III (the maze IV procedure is the same except that the pulmonary vein area is ablated using a circumferential lesion set versus a box lesion set) procedure has met with great success as reported by Washington University and has shown a significant reduction in cerebrovascular accidents and transient ischemic events due to the high success rate of ablating atrial fibrillation and amputating the left atrial appendage.^7^–^10^ In addition, fewer pacemakers have been implanted, and improved atrial transport and sinus node function have been seen.^7^–^10^ The question now becomes what is the best energy source to use when performing the maze procedure.

### Cryothermy Energy

Cryosurgical ablation is defined as the use of a certain temperature below freezing to obtain a specific tissue response.^11^,^12^ The severity of the tissue response and destruction is directly related to the intensity of the freezing temperature.^5^ For example, a minor freezing temperature (tissue temperature higher than −25°C to −30°C) causes only inflammation leaving the potential for the tissue to recover, while severe freezing will actually kill the cardiac muscle cells and results in non-reversible tissue destruction. The mechanism of action of the use of freezing temperatures is that there is direct cell necrosis and vascular stasis which occurs only during the thawing period.^11^,^12^

The use of cryothermy as a method of treating different pathologies has been around for a long time. In 1961, Cooper and Lee developed a device that was capable of cooling liquid nitrogen that could be applied on tissue.^13^ In recent years, a number of devices have been developed based on the Joule–Thomson effect, which is a principle in thermodynamics that describes the change in temperature of a thermally insulated gas as it is forced through a small hole or a porous material. For each gas there is a temperature of inversion above which the change is positive and below which it is negative.^14^ A number of devices developed to use cryothermy are able to achieve the boiling points of the gases which is −189°C for argon and −75°C for nitrous oxide. Heat is transferred from the tissue to the probe forming an ice ball which expands over time, and the myocytes are killed when the tissue reaches a temperature below −25°C. It takes about 2 minutes to achieve these lethal temperatures in order to create a full thickness atrial wall lesion minus a heat sink effect which can occur (in atrial tissue up to about 6–8 mm in thickness).^15^–^17^

The full Cox maze III and IV procedure using cryothermy only was performed early on using an older generation nitrous oxide base platform at Georgetown University and Hadassah University medical center.^4^ The use of cryothermy has evolved, and the creation of complete transmural lesions has been confirmed histologically with argon cryoprobes applied endocardially and epicardially in an experimental sheep model, as compared with the nitrous oxide platform.^7^,^12^ The major advantages of cryothermy are: 1) visual confirmation of transmurality by “ice ball” formation; 2) rapid creation of focal lesion; and 3) low risk of injury to adjacent tissues. The advantage of argon-based cryothermy over nitric oxide is that it can reach temperatures of −140° to −160°C compared with −60°C for nitric oxide, allowing for a much faster and more reliable lesion and the ability to treat thicker tissue.^7^,^11^,^12^

### Radiofrequency Energy

Radiofrequency (RF) energy uses alternating current to heat tissue, creating thermal injury that results in a line of conduction block.^18^–^22^ In unipolar systems, grounding is achieved by an indifferent electrode applied to the skin (usually the back), and current flows from the tip of the RF catheter and resistively heats tissue in contact with the tip. RF energy is delivered to the tissue between the jaws of the clamp at 75 volts and 750 milliamps (mA).^20^ The RF generator monitors voltage, current, temperature, time, and tissue conductance. Energy delivery is continued until tissue conductance between electrodes in the jaws of the clamp decreases and reaches a steady state for 2 seconds.^21^,^22^

Current studies have found that bipolar radiofrequency is superior to unipolar radiofrequency.^19^,^21^ Bipolar RF is able more consistently to create transmural lesions especially when working only with the ablation of the pulmonary veins since the shape of the clamp allows easy placement around the pulmonary veins. Endocardial blood flow has also not been shown to influence the ablation lesion depth.^22^

Microwave and ultrasound energy sources have been used as well in surgical ablation.^23^ However, studies have shown that these energy sources in the current state do not create transmural lesions consistently so the long-term efficacy in achieving a return to sinus rhythm is very low.^20^,^21^ In fact, the Federal Food and Drug Administration (FDA) recently removed its approval for the use of ultrasound in surgical ablation procedures.

## ABLATION PROBES

**Figure 1:**
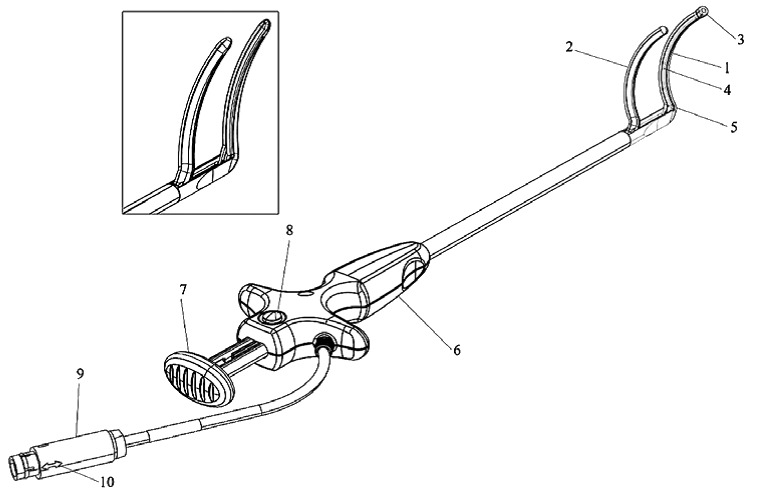
**Atricure^®^Radiofrequency Ablation Probe.** 1, Distal jaw; 2, proximal jaw; 3, attachment tip; 4, electrode; 5, jaw heel; 6, handle; 7, closure lever; 8, release mechanism; 9, connector; 10, connector alignment arrow. Picture provided courtesy of the device manufacturer.

**Figure 2:**
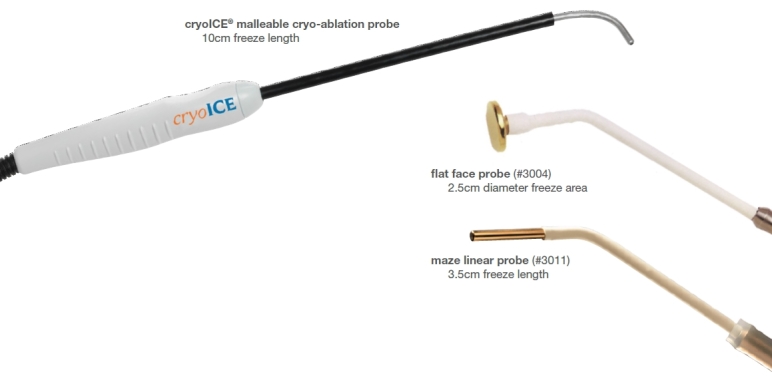
**Atricure^®^CryoFlex Probe 1.** Picture provided courtesy of the device manufacturer.

Table 1 displays the ablation probes that are in use today. The cryothermy probes are produced by Medtronic (Minneapolis, MN, USA), and the radiofrequency probes are produced by Atricure (Schiphol, The Netherlands) (Figure 1).^15^,^16^ The Cardioblate^®^ CryoFlex™ Surgical Ablation System is intended for minimally invasive cardiac surgical procedures, including the treatment of cardiac arrhythmias. The Cardioblate CryoFlex 7-cm, 10-cm, and 10-S probes plus the Cardioblate CryoFlex Clamp and Cardioblate CryoFlex Surgical Ablation Console freeze target tissue and block the electrical conduction pathways by creating an inflammatory response and cryonecrosis. Atricure developed a new cryoprobe that is being used with the nitric oxide platform; the new probe is semi-flexible and can be used to apply all lesions required for the maze procedure. Unlike the CryoFlex, the Cryo1 probe has a defrost feature that facilitates quick removal of the probe from the tissue (Figure 2). All ablation devices may induce complications due to their potential damage the cardiac tissue.^15^,^16^

**Table 1:**
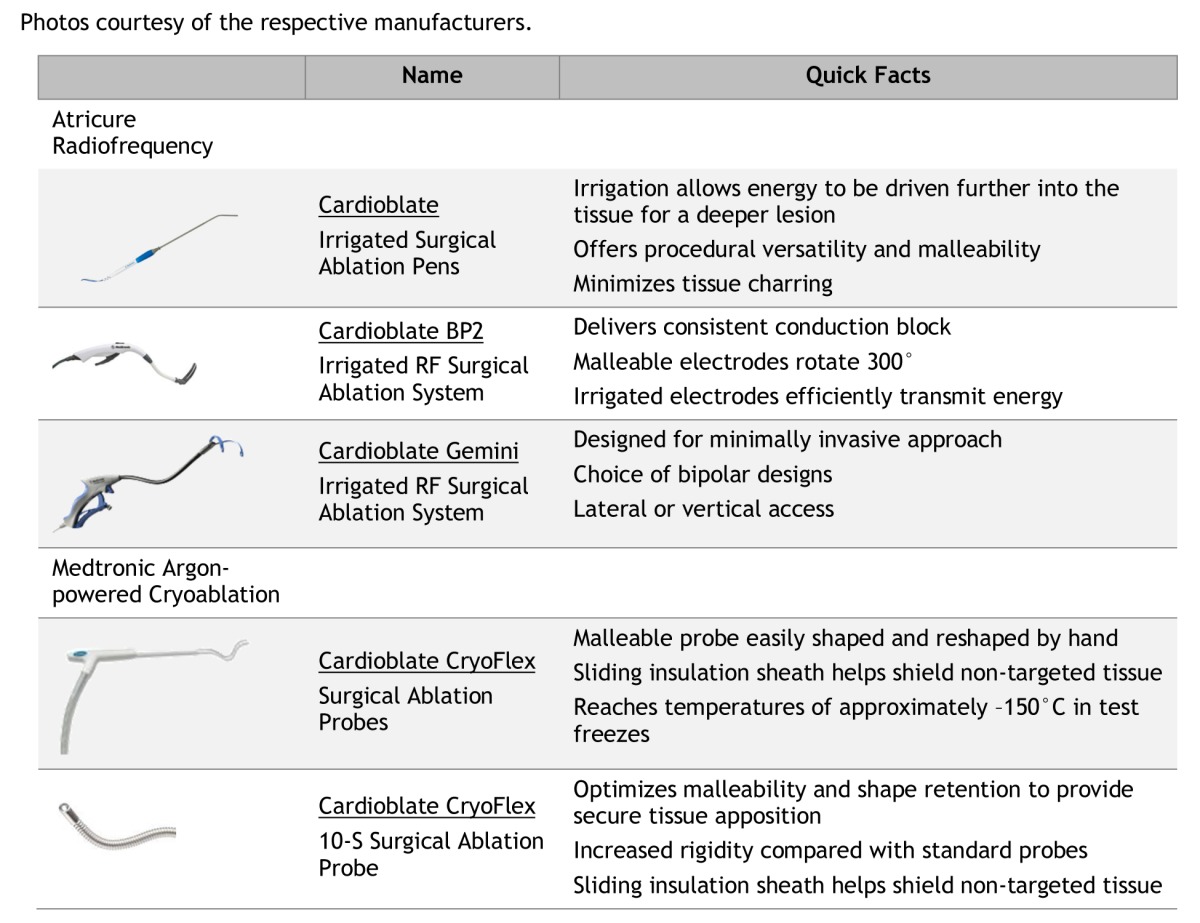
**Ablation Devices. ** Photos courtesy of the respective manufacturers.

**Figure 3:**
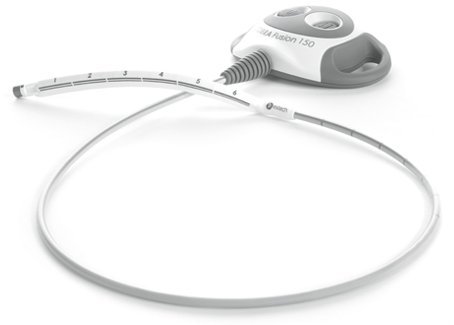
**Estech COBRA Fusion™ System.** Picture provided courtesy of the device manufacturer.

Estech (San Ramon, CA, USA), a leader in minimally invasive and endoscopic cardiac ablation, has recently gained FDA conditional Investigational Device Exemption (IDE) approval to start a trial study (Figure 3). The IDE trial has been designed to evaluate the treatment of atrial fibrillation (AF) utilizing a multiple temperature-controlled radiofrequency (TCRF) device used to treat non-paroxysmal AF. The Estech device is called the COBRA^®^ Surgical System. Up to 15 centers in the United States and Europe will participate in the trial, which is intended to enroll over 100 cardiac surgery patients.^17^

## SURGICAL APPROACHES

In the past decade, several surgical ablation approaches to treat atrial fibrillation as a stand-alone or concomitantly with other cardiac surgical procedures were developed.^1^–^6^,^24^–^31^ The use of the heart–lung machine is required to perform the Cox maze procedure in its current form and with the available ablation devices.^1^–^3^ It can be performed as a full sternotomy or minimally invasive procedure.^1^-^6^,^24^–^31^ If other procedures such as valve repair/replacement or coronary bypass are to be performed concurrently with the Cox maze procedure, then the standard open chest approach is likely to be used; however, when atrial septal defect closure, mitral valve repair or replacement, and tricuspid valve surgery are required, a minimally invasive right mini-thoracotomy can be applied.^1^–^6^,^24^–^31^ The overall operative risk is low in morbidity and mortality and might be impacted by the individual’s specific health conditions.^8^–^10^

### Cox Maze Procedure by Mini-Thoracotomy

Despite the proven efficacy of surgical ablation, a fraction of patients and referring physicians are still unwilling to tolerate sternotomy for an arrhythmia-corrective procedure.^18^ This has led to the development and evaluation of minimally invasive surgical ablation procedures. The term “minimally invasive” as it applies to cardiac surgery incorporates a combination of small, sternum-sparing incisions, alternative cannulation techniques, modified instruments, thoracoscopic visualization, and robotic assistance. Given that the Cox maze III has a proven track record for sinus restoration and improved quality of life, surgeons must be careful in performing a minimally invasive procedure with inferior results, particularly in those with non-paroxysmal atrial fibrillation. The two current major strategies are a complete Cox maze III using minimally invasive approach and totally endoscopic left-sided surgical ablation approach.

## PROCEDURE

Briefly, our approach is described here. We use double-lumen endotracheal intubation for selective right-lung deflation. The patient is placed in the supine position with a single towel roll underneath the posterior right rib cage. The right shoulder is abducted and arm is flexed and secured to an arm bar. A 5–6 cm incision is placed beneath the right breast, and the right chest is entered through the fourth or fifth intercostal space. CO_2_ insufflation is used. The pericardium is opened approximately 2 cm anterior to the phrenic nerve. The pericardium is secured to the chest wall for retraction, and umbilical tapes are placed around the superior vena cava (SVC) and inferior vena cava (IVC).

The right femoral artery and femoral vein are exposed through a 3 cm oblique groin incision, heparin is administered, and vessels are cannulated. The femoral vein cannula is positioned into the IVC just below the level of the diaphragm as confirmed by transesophageal echocardiography.

For the cross-clamp technique, cardiopulmonary bypass (CPB) is instituted, and the left ventricle is vented through the right superior pulmonary vein. A cardioplegia cannula is placed in the ascending aorta, and the aorta is cross-clamped through a stab wound in the right lateral chest. The heart is arrested, and umbilical tapes are secured around the SVC and IVC. The right atriotomy is made parallel to the septum, and the following right-sided lesions are created: 1) a lesion from the lower end of this incision to the tricuspid valve; 2) a lesion to the tricuspid annulus anterior to the membranous interatrial septum; and 3) lesions on the right atrial free wall to the inferior vena cava, superior vena cava, and atrial septum. Alternatively, three 5 mm incisions with purse-string sutures can be placed and serve as access points to complete a full right-sided Cox maze III lesion set.^2^

Next the left atrium is entered through an atriotomy in the interatrial groove. The left-sided lesions include: 1) superior and inferior lesions encircling the right and left pulmonary veins to the left atrial appendage suture line; 2) a posterior lesion to the level of the mid-mitral valve annulus; and 3) an epicardial coronary sinus lesion. The left atrial appendage is oversewn from the inside with 4-0 monofilament suture. Air is evacuated using carbon dioxide insufflation and de-airing maneuvers which include rotating the table to the far left and repeatedly inflating the left lung. The heart is rewarmed with warm blood cardioplegia. The aortic cross-clamp is then released, and during the remaining rewarming phase the two right atriotomies are closed with 4-0 monofilament suture. Temporary atrial and ventricular pacing wires are placed, and the patient is weaned from CPB. The cannulas are removed, the heparin is reversed, and thoracotomy and groin incisions are closed in a standard fashion.

More recently, we have transitioned to a fibrillating heart technique without cross-clamp. Here, patients are cooled to 30–32°C with pump flow rates between 2.0 and 2.5 L/min per square meter and mean arterial pressures between 50 and 60 mmHg. Ventricular fibrillation is induced prior to opening the left atrium. Two suction catheters are placed in the atrium. Upon completion of the left and right lesions, the patient is rewarmed, defibrillated using external pads (Medtronic, Minneapolis, MN, USA), and weaned off CPB.

Importantly, to reduce the potential for stroke and vascular complications secondary to femoral cannulation, preoperative CT angiography was routinely obtained for comprehensive assessment of aortic and peripheral arterial anatomy. This procedure can be performed as a concomitant procedure at the time of mitral valve surgery. The results expected from this technique are in the process of publication. A series of 104 patients treated for non-paroxysmal AF (80% long-term persistent AF) has yielded promising results. At 2 years 80% of the patients are in sinus rhythm with no class I/III antiarrhythmic drug therapy, and 91% are in sinus rhythm regardless of antiarrhythmic treatment. At 5 years the freedom from any atrial arrhythmia is 81%. All patients were followed with 24 h Holter monitoring.

### Video-Assisted Surgical Ablation

The thoracoscopic surgical ablation procedure was first based on pulmonary vein isolation (PVI) with additional lesion sets but now includes more extensive left-sided lesions. Epicardial ablative devices have allowed for the evolution of off-pump, thoracoscopic approaches. In a landmark finding, Haissaguerre et al. found that the pulmonary veins were the major source of atrial fibrillation ectopic foci.^27^ This led to the first bilateral PVI with left atrial appendage (LAA) exclusion using bilateral thoracoscopic mini-thoracotomies.^26^ Minimally invasive ablation via bilateral mini-thoracotomies for paroxysmal AF is associated with 80.8% freedom from AF at 1 year.^25^ Thoracoscopic bilateral PVI with LAA exclusion has also been described for treatment of lone AF refractory to catheter ablation.^30^,^31^ This was extended to include PVI, LAA exclusion, and ablation of ganglionic plexus (GP) and ligament of Marshall.^1^–^6^,^32^ Bilateral PVI, LAA, and GP ablation at 6 months was found to be more effective for paroxysmal AF; 86.7% of patients with paroxysmal fibrillations were in normal sinus rhythm and 71.7% were both in normal sinus rhythm and off antiarrhythmic drugs (AADs).^28^ Less so was observed for the patients with persistent atrial fibrillation, of whom 56.3% were in normal sinus rhythm and 46.9% both in normal sinus rhythm and off AADs.^28^ As to long-standing persistent cases, 50% were in normal sinus rhythm and 31.9% were also off AADs.^28^ The “Dallas lesion” added further left atrial linear ablation lines.^29^

Clinical and experimental electrophysiological studies have found ectopic impulses originating from the autonomic ganglionic plexus in epicardial fat adjacent to the atrial pulmonary vein interface to be a source of arrhythmias.^33^–^36^ To address this, GP ablation may be performed as an adjunct to surgical ablation procedures. A prospective randomized trial of 67 patients demonstrated improved freedom from AF with the addition of ganglion plexus ablation to PVI (85.3% versus 60.6% freedom from AF) at 4.3-month follow-up.^37^ Similarly, comparison of patients with GP ablation with maze versus a case-matched control cohort found significantly higher freedom from AF at 1 year (90% versus 50%).^38^

At mid-term follow-up, among patients with long-standing persistent AF undergoing thoracoscopic PVI and GP ablation, 92.7% of patients treated with irbesartan were in sinus rhythm compared with 67.5% in a control group.^39^ These results suggest that the efficacy of minimally invasive surgical ablation may be augmented using adjunctive medical treatments.^39^

Most recently, Weimer et al. presented a series of thoracoscopic left-sided bipolar RF ablations on 89 patients with 35% paroxysmal and 65% persistent/long-standing persistent symptomatic AF.^7^ Their lesion set included PVI with upper and lower pulmonary vein connecting lines, a lesion to the LAA and on the left atrial roof to the aortic valve non-coronary sinus, as well as LAA stapler exclusion. They report 90% 2-year freedom from AF and off AAD, with no mortality and no stroke events.^40^

There are some caveats with regards to LAA isolation. The limitation of the right-sided thoracoscopic approach is the inability to exclude the left atrial appendage, although new devices may allow for endocardial occlusion. Also, of note, the Left Atrial Appendage Occlusion Study (LAAOS) trial revealed that a significant proportion of endocardial LAA closures using an encircling technique or a running suture as well as staple exclusion recannulated when assessed by echocardiography (55% versus 28%).^41^ This suggests that complete LAA amputation may be superior to suture ligation or staple exclusion.

### Robotic-Assisted Surgical Ablation

Loulmet and colleagues first described robotic PVI using a flexible microwave probe through the left chest.^42^ This was extended to microwave ablation via a right mini-thoracotomy with groin cannulation on cardiopulmonary bypass.^43^ More recently, Cheema et al. report successful robotic argon cryoablation with femoral cannulation for completion of left-sided surgical ablation lesions and endocardial LAA exclusion.^44^

In summary, the field of surgical ablation for atrial fibrillation is rapidly expanding. In this paper, different energy sources currently available when performing a surgical ablation procedure for atrial fibrillation were discussed. Based on the published literature and our own experience, the two most prominent energy sources currently used are cryothermy and bipolar radiofrequency. Three surgical approaches for the performance of the atrial fibrillation ablation procedure were also discussed. Median sternotomy remains the approach most commonly used; however, the use of a minimally invasive approach is becoming more refined thus offering patients a viable alternative approach to the performance of the procedure. The use of robotic technology in performing the surgical ablation procedure is one of the newest platforms for the performance of the ablation procedure so was briefly discussed.

## CONCLUSION

The surgical treatment for atrial fibrillation has changed over the past decade. Today the vast majority of the procedures are being performed using alternative energy sources to create the lesions. In the field of surgery for stand-alone atrial fibrillation a lot of beating-heart procedures are being performed, with minimal success, however, especially in patients with persistent and long-term persistent atrial fibrillation.

## Abbreviations

AAD: antiarrhythmic drugs;
AF: atrial fibrillation;
CPB: cardiopulmonary bypass;
FDA: Federal Food and Drug Administration;
GP: ganglionic plexus;
IDE: Investigational Device Exemption;
IVC: inferior vena cava;
LAA: left atrial appendage;
PVI: pulmonary vein isolation;
RF: radiofrequency;
SVC: superior vena cava;
TCRF: temperature-controlled radiofrequency.
